# Mid-term results of giant cell tumours with pathologic fractures around the knee: a multicentre retrospective study

**DOI:** 10.1186/s12891-022-06005-1

**Published:** 2022-12-05

**Authors:** Liming Zhao, Jiapei Chen, Yongcheng Hu, Zhaoming Ye, Kun Tao

**Affiliations:** 1 Department of Joint Surgery, Ningbo No.6 Hospital, No. 1059 East Zhongshan Road, Ningbo, 315040 Zhejiang Province China; 2 Langfang Health Vocational College, Siguang South Road, Langfang, 065000 Hebei Province China; 3 Department of Orthopaedic Oncology, Tianjin Hospital, No. 406 Jiefang South Road, Tianjin, 300211 China; 4 Department of Orthopaedic Oncology, The Second Affiliated Hospital of Zhejiang University, No. 1511 Jianghong Road, Hangzhou, 315040 Zhejiang Province China

**Keywords:** GCT, pathologic fracture, classification, multicentre study

## Abstract

**Objective:**

The aims of this work are to present a classification of “complex fracture” and “simple fracture”, to compare their features, treatments and prognosis in patients with giant cell tumour with pathologic fractures around the knee, and to determine the best surgical method for patients who have giant cell tumour around the knee with different degrees of fracture.

**Methods:**

Data from 130 patients with pathologic fractures from giant cell tumour around the knee who underwent surgical treatment from March 2000 to November 2015 at 6 institutes around China were collected and analysed. A multicentric study design was used to explore the epidemiological features and to compare differences in the surgical procedures and prognosis of the two fracture groups. The mean age at diagnosis was 37.1 years old (range, 13-77 years). The median follow-up was 126.5 months, ranging from 68 to 370 months.

**Results:**

The general clinical and imaging features of the groups of patients with simple and complex fractures, namely, sex, age, the lesion site, living or working environment, eccentric growth patterns, Campanacci grading system, and duration of symptoms before treatment, showed varying degrees of differences, but with no statistical significance (*p* > 0.05). The incidence rate of surrounding soft tissue mass was 35.2% (32/91) in the group with simple fractures, whereas it was 87.2% (34/39) in the group with complex fractures, which showed a significant difference (*p <* 0.05). Wide resection and reconstruction with joint replacement were performed more often in patients with complex fractures (61.5%, 24/39). Intralesional procedures were performed more often in patients with simple fractures (56.0%, 51/91). The difference showed significant differences (*p <* 0.05). The local recurrence rate was 17.6% (16/91) in the group with simple fractures, whereas it was 10.3% (4/39) in the complex fracture group, showing a significant difference (*p <* 0.05). A total of 2.3% of patients (*n =* 3,3/130) developed a skip lesion. The complication rates were 4.6% (4/87) and 14.7% (5/34), respectively, in the two groups with simple or complex fractures, showing a significant difference (*p <* 0.05). The mean MSTS and TESS scores with simple fractures were 26.6 (range, 13–30) and 84.1 (range, 29-100), respectively, whereas the mean scores in the group with complex fractures were 25.5 (range, 18–30) and 78.3 (range, 30-100), respectively, also showing a significant difference (*p <* 0.05).

**Conclusion:**

Our classification of “simple fracture” and “complex fracture” could guide decisions regarding the best surgical method for lesions in patients who have giant cell tumour around the knee with different degrees of fracture.

## Background

Giant cell tumours (GCTs) of bone are intermediate, locally aggressive tumours that usually produce osteolysis and cause cortical breach [1–4]. Even though GCT is not a deadly disease, it may weaken the bone sufficiently to cause fracture. Of particular note is that pathologic fractures occurring at first presentation reportedly vary between 9 and 30% of all patients [5–8]. Nearly more than 50% of GCTs develop in the region around the knee [1, 4, 7–11]. Adjacent tissue contamination and articular surface damage may occur due to pathologic fracture, with different degrees increasing the surgical difficulty and risk of recurrence to different extents [4, 11, 12]. Therefore, concerns related to the degree of pathologic fracture have real-world significance.

The accepted classification systems are mainly based on the histological and radiological features of GCT. The histological grading system presented by Jaffe et al. attempted to stratify these tumours into three classes: benign, aggressive or malignant [13]. Although Dahlin et al. simplified the system by dividing them only into benign and malignant lesions, both systems had poor correlation with clinical course and imaging features [14]. Later, Enneking expanded the grading for benign tumours into three classes and malignant tumours into three classes and five stages, which was associated with the clinical course but lacked specificity [15]. Due to these various shortcomings and insufficiencies, the grading systems mentioned above have already been abandoned. The most widely used grading system at present is that developed by Campanacci et al., who classified GCT into three classes solely based on the radiological appearance, revealing that GCT with fractures were all grade 2 or grade 3 lesions [7]. However, the degree of fracture was not discussed. Huber and Gerber recommended that patients with grade 1 or grade 2 GCTs should undergo intensive treatment until the fracture is healed, followed by curettage, whereas immediate open biopsy and wide resection have been advised for patients with grade 3 pathologic fractures [16]. Heijden et al. retrospectively reviewed 48 patients with pathologic fractures, and their results showed that wide resection should be considered with soft tissue extension, in cases of complex fracture, or when structural integrity cannot be regained after reconstruction [17]. However, he did not indicate the definition or features of simple fracture and complex fracture. Several authors agreed that different surgical methods should be adopted depending on whether the lesions have intra-articular involvement with the fracture [1, 7, 18–21].

Previous studies have indicated that pathologic fracture is a controversial factor in selecting the most reasonable surgical method and resection rang e[1, 7, 18, 19, 21–24]. This suggests that individual treatment is needed for pathologic fractures of varying severity. However, different degrees of fractures are not stratified by radiologic diagnosis. It is equally disappointing that few articles have addressed the type and severity of pathologic fractures. Due to the lack of detailed judgement criteria for pathologic fractures, surgeons struggle to make the best treatment decision. In reality, pathologic fracture is a clinical concept rather than a radiologic concept. We consider it a complex fracture if the pathologic fracture causes increased surgical difficulty. Accordingly, we need to pay very close attention to the diagnosis and treatment of GCT patients with pathologic fractures.

The previous reports that have ignored the imaging features of pathologic fractures due to GCTs were generally retrospective single-centre analyses, and the lesions were located in various parts of the body [9–11, 14, 25]. Therefore, a multicentric study featuring a large sample of patients with a single lesion around the knee is needed. We aimed to (1) determine a classification system for “complex fracture” and “simple fracture” to aid in the diagnosis of pathologic fracture due to GCT around the knee; (2) compare the differences in the epidemiological, clinical and imaging features between the two groups with complex fracture and simple fracture at presentation; and (3) describe the differences in the selection of the surgical procedure and prognosis between the complex fracture group and the simple fracture group.

## Materials and methods

The giant cell tumour team of China (GTOC) was founded in July 2010, and over the years, it has evolved into a cooperative institution with wide recognition around China, including eleven centres in different provincial capitals [25, 26]. The original aim of the GTOC was to undertake research limited to GCTs around the knee through retrospective analyses. Even more importantly, these studies will lay the foundation for prospective studies in the future. Patients with a diagnosis of GCT were recruited from six musculoskeletal tumour centres that had previously participated in our GTOC [26, 27].

From March 2000 to November 2015, we retrospectively reviewed all 553 patients with histologically confirmed lesions of GCT of the proximal tibia or distal femur, of whom 180 had presented with pathologic fracture. A total of 27 patients who underwent primary treatments at locations other than the six centres of the GTOC were excluded. We also excluded 21 patients who had insufficient clinical and imaging data or who were lost to follow-up. In addition, we excluded 2 patients who underwent amputation during the primary surgery. One patient had a confirmed period of 2 years without treatment after diagnosis, and the other patient suffered from severe infection of the surrounding soft tissues (Chart 1).

Intralesional procedures were the most common clinical treatment options, namely, intralesional curettage and excision with internal fixation, which is based on focal features using plates, screws only or intramedullary nails. For these intralesional procedures, a sufficiently wide cortical window was created for visualization of the tumour cavity and excision of the pseudocapsule, likely through soft tissue extension. We removed the tumour bulk with curettes to reach normal-appearing bone, and the tumour cavity was further enlarged in all directions with a high-speed burr. If necessary, subchondral bone can also be ground away. Sterile water was used to irrigate the bone defect. Our GTOC then used varying types of adjuvants to pre-process the cavity for a while, followed by cement reconstruction, morselized or/and structural grafts with autologous or/and allogenic bone, or a combination of them.

Reconstructions performed with resection or cement provide immediate stability. Patients were encouraged to perform early active motion and weight-bearing exercises. Those who did not undergo reconstruction were kept non-weight-bearing with or without a cast for at least 6 to 8 weeks, followed by gradually increasing motion as radiographs showed an ideal outcome. There were only 2 patients who received external beam radiation therapy preoperatively and postoperatively because of the high-grade malignancy of GCT cells whose pathological diagnosis was still considered as GCT.

Due to the poorly unified management of multicentre studies, follow-ups were not routinely scheduled except at three centres. Plain radiographs of the local area were obtained at every follow-up. In cases of suspicious local recurrence, MRI and CT were recommended. For patients with a high risk of recurrence, plain radiography or CT of the lung were recommended. For our calculations, we needed to include the patients who experienced clinical symptoms for less than 1 month with those who experienced symptoms for one month.

Patients were mainly followed-up through outpatient review (*n =* 83, 63.8%), with some completing telephone review (*n =* 47, 36.2%). The median follow-up duration was 126.5 months, ranging from 68 to 370 months. We recorded the local recurrence rate (*n =* 20, 15.4%), complication rate (*n =* 9, 6.9%), metastasis rate (2.3%, 3/130), and mortality rate (3.1%, 4/130) (one died from late syphilis, one had a metastatic lesion in the lung after surgery with joint replacement for 23 months and died due malignant transformation 3 months later, one died due to lung metastasis only, and one died from pulmonary heart disease).

The Musculoskeletal Tumour Society (MSTS) score and Toronto Extremity Salvage Score (TESS) were obtained to assess functional outcome of the affected extremity.

We separated the patients into two different groups in accordance with our classification criteria of pathologic fractures; the determination of whether the fracture was simple or complex was left to the discretion of the surgeon and based on the surgical difficulty. Professors of tumours of bone and soft tissue at the GTOC generated brief and concise unified differentiation criteria for pathologic fracture, which was assessed based on the degree of gross fracture displacement and so the risk of surrounding tissues contamination.

**Fig. 1:**
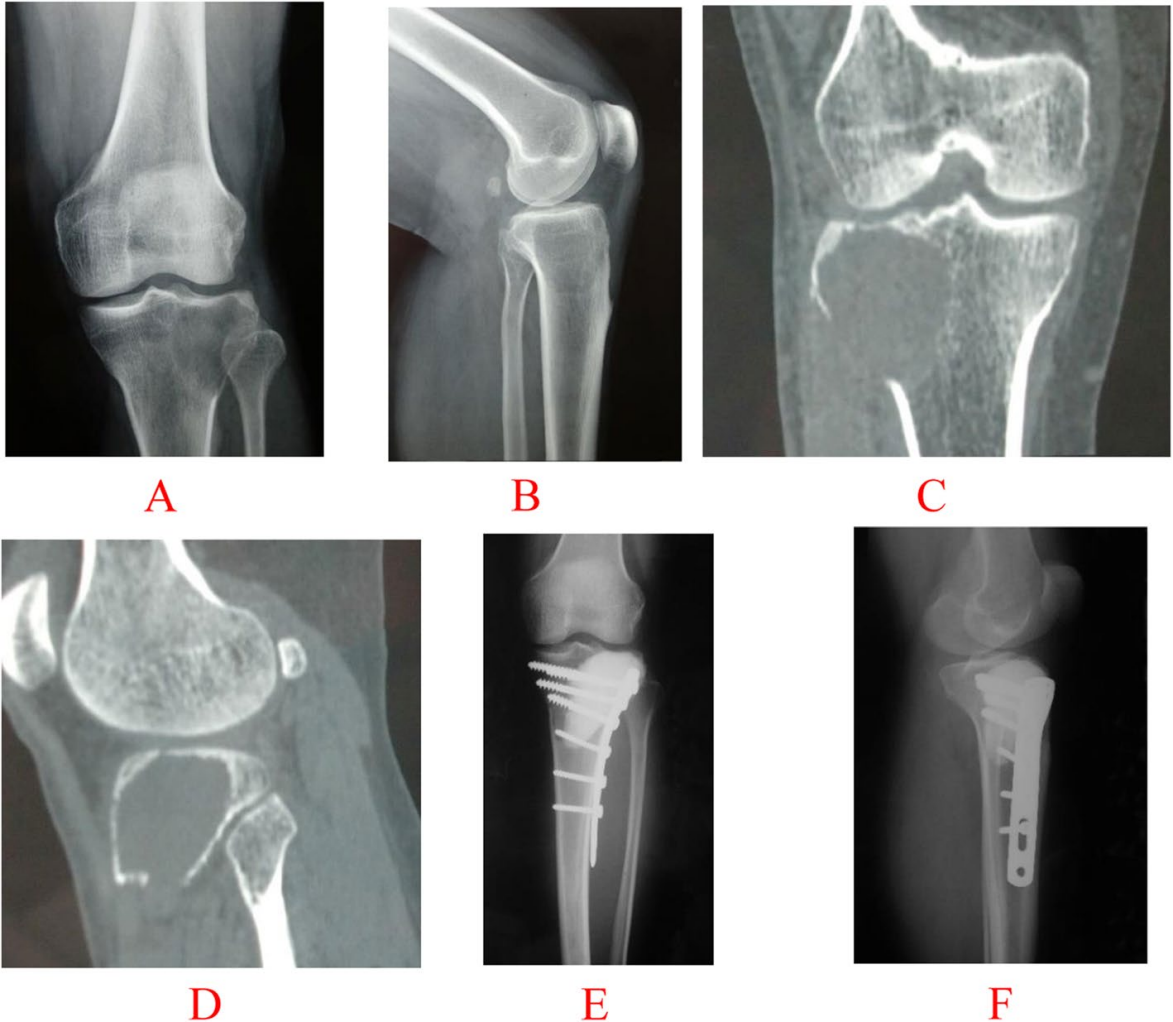
A 51-year-old male with a simple pathological fracture through GCT of the proximal tibia was treated with intralesional procedure. **A**, **B** Anteroposterior (AP) and lateral preoperative radiographs show the fracture is basically aligned. **C**, **D** Coronal and sagittal CT images show it involves extra-articular fracture and articular fracture with an broken joint surface, whose distance of fracture displacement less than 2 mm. **E**, **F** Anteroposterior (AP) and lateral postoperative radiographs made immediately after curettage, reconstruction with bone cement and internal fixation

**Fig. 2:**
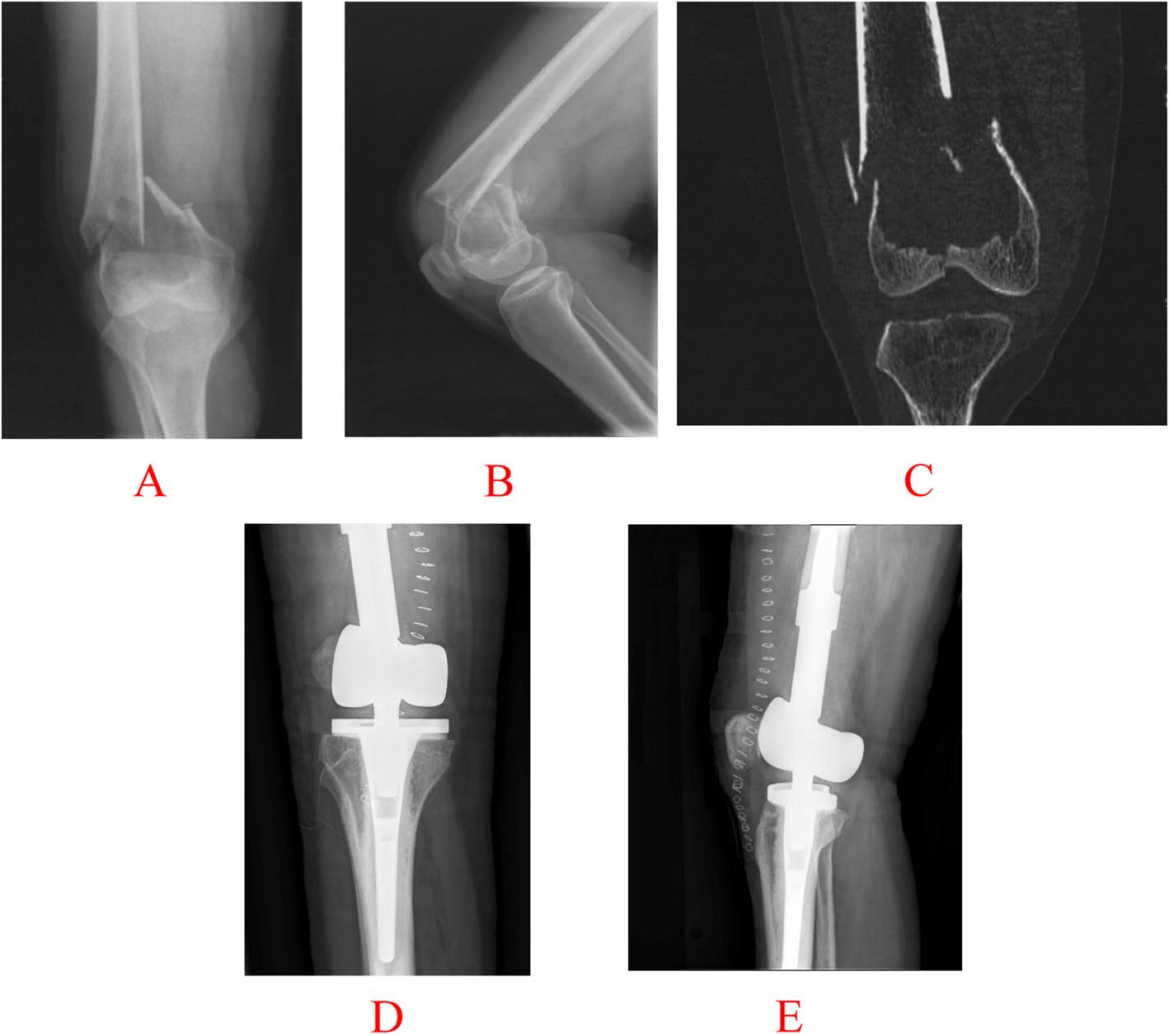
A 39-year-old female with a complex pathological fracture through GCT of the distal femur was treated with resection. **A**, **B** Anteroposterior (AP) and lateral preoperative radiographs show bicondylar displaced fracture through a large lytic defect with intraarticular extension. **C** A coronal CT image shows bone lysis with an absence of the integrity of joint surface, and the distance of fracture displacement is more than 2 mm. **D**, **E** AP and lateral postoperative radiographs made immediately after resection reconstructed with prothesis

Simple fractures: (1) Intra-articular fractures with an unbroken joint surface, or the distance of fracture displacement less than 2 mm; (2) Extra-articular fractures with no or minor displacement, and the fracture is basically aligned (Fig. 1). Complex fractures: (1) Intra-articular fractures damage the integrity of the joint surface, and the distance of fracture displacement more than 2 mm; (2) Comminuted fractures or massive gross fracture displacement (Fig. 2).

Recurrence-free survival rates were assessed using Kaplan–Meier survival analysis. Independent t tests were performed to assess group differences in continuous variables. To assess group differences in ordinal variables or continuous variables with nonnormal distributions, the Mann–Whitney test was used. The continuity adjusted Chi-square test or Fisher’s exact test was used to compare categorical variables. Functional outcomes (MSTS and TESS scores) for both groups were compared with an unpaired t test. For all statistical tests, an outcome of *p <* 0.05 was considered significant. SPSS 19.0 (SPSS Inc., Chicago, IL, USA) was used to perform all statistical analyses.

## Results

Among all included patients (*n =* 130), there was a slight predominance of female patients (52%). The mean age at diagnosis was 37.1 years old (range, 13-77 years). In these patients, 42.3% (*n =* 55) occurred in the left knee joint, and 57.7% (*n =* 75) occurred in the right knee joint. A total of 61.5% of patients (*n =* 80) located in the distal femur, and 38.5% of patients (*n =* 50) located in the proximal tibia. In the whole study group, 10.8% of the tumours (*n =* 14) were found to exhibit eccentric growth, which was remarkably less than the proportion of tumours found to exhibit non-eccentric growth. When the grading system developed by Campanacci et al. was used, GCTs with fractures were all grade 2 (*n =* 34, 26.2%) or grade 3 (*n =* 96, 73.8%) lesions. The period of time that the patients experienced symptoms before treatment lasted for a median of 5.5 months (range, 1 ~ 120 months). Regarding the patients’ living and working environments, 70.8% (*n =* 92) lived in villages, and 29.2% (*n =* 38) lived in cities and towns.

The general clinical and imaging features of the patients, namely, sex, age, the lesion site, living or working environment, eccentric growth patterns, Campanacci grading system, and duration of symptoms before treatment, showed varying degrees of differences between the two groups with simple fractures and complex fractures, but with no statistical significance (*p >* 0.05) The incidence rate of surrounding soft tissue mass was 35.2% (32/91) in the group with simple fractures, whereas it was 87.2% (34/39) in the complex fracture group, which showed a significant difference (*p <* 0.05) (Table 1).

**Table 1:** Comparison of clinical and imaging features between the simple pathological fracture group and the complex pathological fracture group

Variable	Fracture		Total	Statistics
	Simple	Complex		*P* value
Gender				
Male	41	21	62	0.36
Female	50	18	68	
Patient age at diagnosis (years)*	36.7	38.0		0.65
Living environment				
Village	64	28	92	0.87
Cities and towns	27	11	38	
Knee joint				
Left	39	16	55	0.85
Right	52	23	75	
Distal femur	56	24	80	1.00
Proximal tibia	35	15	50	
Eccentric growth				
Yes	84	32	116	0.08
No	7	7	14	
Campanacci system				
Grade-2	26	8	34	0.34
Grade-3	65	31	96	
Soft tissue mass				
Absent mass	59	5	64	0.03*
Developing mass	32	34	66	
Duration of symptoms (months)				
≤ 6	57	27	84	0.47
>6	34	12	46	
Surgical methods				
Intralesional procedures	51	15	66	0.04*
Resection with joint replacement	40	24	64	
Choices of internal fixation				
Plate	31	14	45	0.43
Screws only	2	0	2	
Intramedullary nail	3	0	3	
Recurrence				
Yes	16	4	20	0.03*
No	75	35	110	
Complication				
Yes	4	5	9	0.04*
No	87	34	121	
Functional outcome (median scores)				
MSTS	26.6	25.5		0.02*
TESS	84.1	78.3		0.03*

*Values are expressed as mean, *P < 0.05

A total of 50.8% of patients(*n =* 66) accepted the surgery of intralesional procedures, including intralesional curettage (*n =* 16) and excision with fixation (*n =* 50). Resections with protheses were performed in 49.2% of patients (*n =* 64). Wide resection and reconstruction with joint replacement were performed more often in patients with complex fractures (61.5%, 24/39). Intralesional procedures were performed more often in patients with simple fractures (56.0%, 51/91). The difference showed significant differences (*p* < 0.05) (Table 1).

**Fig. 3:**
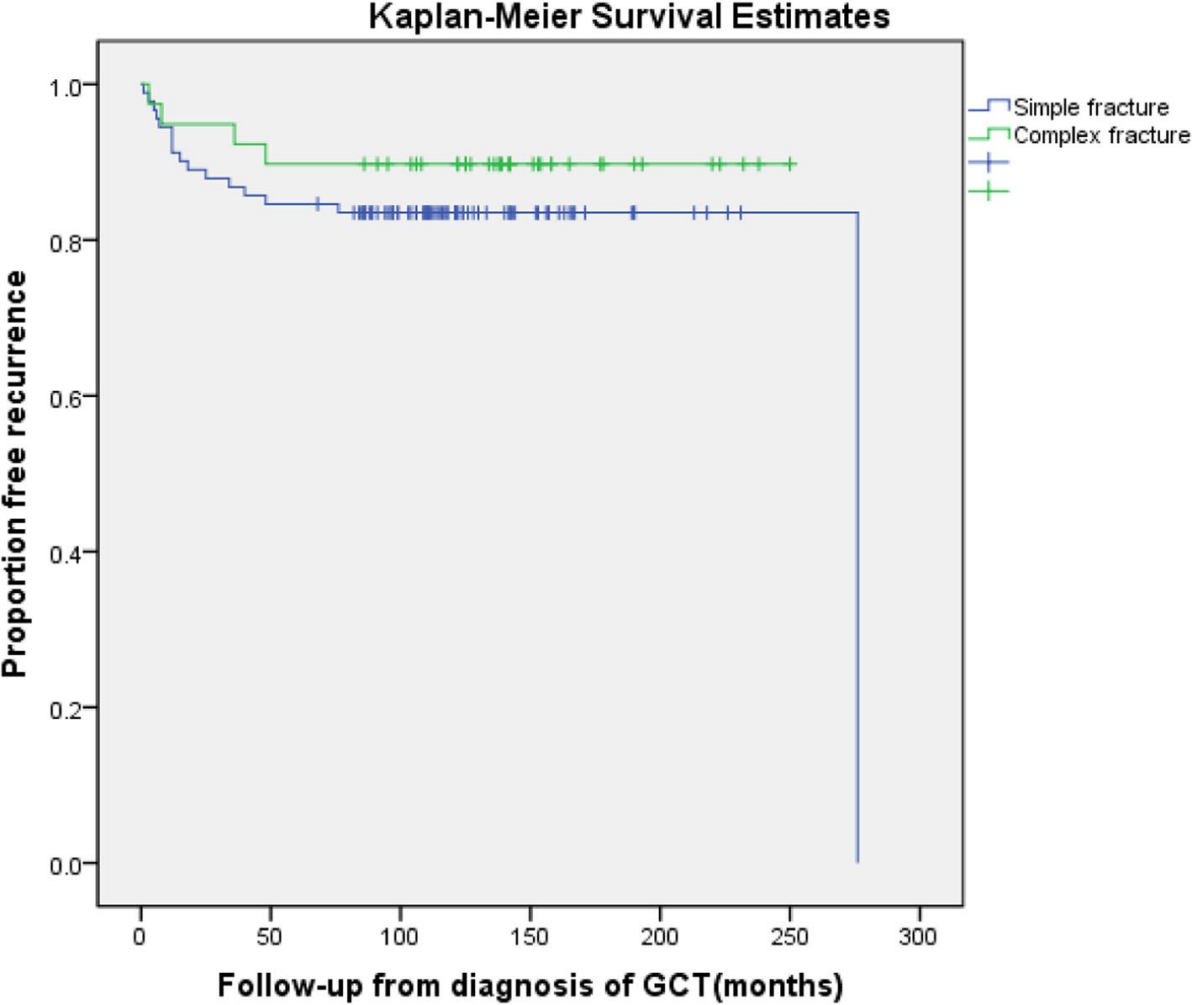
Recurrence-free survival (RFS) between simple fractures group and complex fractures group (with 95% confidence interval), the RFS was lower in group with simple fractures than group with complex fractures (*p* < 0.05)

The local recurrence rate was 17.6% (16/91) in the group with simple fractures, whereas it was 10.3% (4/39) in the complex fracture group, showing a significant difference (*p <* 0.05) (Table 1) (Fig. 3). The mean time to recurrence was not significantly different between the groups (*p* > 0.05): 44.0 months (range, 1–276 months) in the simple fracture group and 23.8 months (range, 3–48 months) in the complex fracture group. The local recurrence rate was 27.3% (18/66) after intralesional procedures and 3.1% (2/64) after resection reconstructed with joint replacement, which showed a significant difference (*p <* 0.05) (Table 2).

**Table 2:** Giant cell tumour of bone treated with curettage versus resection

Surgical treatment	Intralesional procedures	Resection with joint replacement	P value
Total patients (*n =* 130)	66	64	0.01*
Local recurrence	18	2	

The surgical treatments for all first and second local recurrences are summarized in Table 3. Curettage and resection with replacement were performed in most recurrent patients (16/20). One patient developed a lesion that recurred with infection and eventually underwent amputation. A total of 2.3% of patients (*n =* 3,3/130) developed a skip lesion and underwent lung resection but then experienced local recurrence. During follow-up, we were informed that 4 patients from our database ultimately died. The complication rates were 4.6% (4/87) and 14.7% (5/34), respectively, in the two groups with simple or complex fractures, showing a significant difference (*p <* 0.05). The types of complications and relevant treatments are listed below (Table 4).

**Table 3:** Methods of treatment for patients with recurrence between groups with simple fractures and complex fractures

Treatment	Simple fracture (*n =* 16)	Complex fracture (*n =* 4)
Curettage	7	1
Resection with prosthesis	6	2
Amputation	1	0
Resection in lung and local recurrence	2	1

**Table 4:** The diversities of complications and relevant treatments

Type	Quantity	Treatment	Outcome
Infection	5	Anti-inflammation or local revision	cured
Fracture	2	Curettage	cured
Deep Vein Thrombosis	1	Anticoagulant therapy-	cured
Severe Deformity	1	Receive no treatment	unimproved

At the latest follow-up date, the mean MSTS and TESS scores of the group with simple fractures were 26.6 (range, 13–30) and 84.1 (range, 29-100), respectively, whereas the mean scores of the group with complex fractures were 25.5 (range, 18–30) and 78.3 (range, 30-100), respectively. The functional outcomes of the MSTS scoring system and TESS scoring system showed obviously significant differences (Table 1).

## Discussion

GCT is most commonly found in the epi-metaphyseal region of long bones. Numerous studies have reported a slight female predominance, with a peak incidence in young adults aged 20-50 years [4, 7, 9–11, 16, 26, 27]. The results of patients with fractures in the GTOC was in complete accordance with the reported literatures. The widely accepted consensus of most authors is that pathologic fractures cause adjacent tissue contamination, causing the development of a surrounding tissue mass [3, 4, 21, 28–32]. Our study is not only in accord with the reported literatures, but also indicates that soft tissue mass increases with the aggravation of complexity of fractures.

Metastases after GCT of bone are relatively rare, occurring in only 1-4% of patients [4, 7, 10, 20, 24, 31, 33]. In our research, the overall metastatic rate was 2.3% (3/130). This suggests that fractures do not increase the risk of metastasis. Although GCTs have rarely been reported to metastasize, due to their potential for aggressiveness, joint salvage operations are not often considered. GCT, as an osteolytic bone tumour, has been reported to be a relatively frequent complication of pathologic fractures [4, 10, 11, 34]. It is also commonly believed that pathologic fractures are associated with a higher recurrence risk [4, 11, 21, 26, 34, 35]. Thus, resection has been the preferred primary treatment for GCTs with pathologic fractures [3, 20, 21, 36]. However, a few studies could not confirm that pathologic fractures are a risk factor for local recurrence [21, 34, 35]. Furthermore, it has been reported that articular resection may result in functional impairment [3, 9, 27, 37]. These findings have motivated further studies to investigate the indications of surgery for GCT with pathologic fractures. Deheshi et al. compared the recurrence and functional outcomes after curettage for patients both with and without pathologic fractures, and they found that the outcomes were similar [6]. Jeys et al. evaluated the treatment methods for different types of fractures and concluded that curettage can be a safe method for cortical breach, but discrete fractures very often require resection [27]. There was a consensus found in our research: The significant results of our study revealed that resection and reconstruction with joint replacement is performed more often in patients with complex fractures, while joint salvage (curettage and excision with internal fixation) is performed more often in more patients with simple fractures. Our results also showed that the local recurrence rate was significantly higher in the group with intralesional procedures than in the group with resection reconstructed with joint replacement. So, the Kaplan–Meier survival curves practically based on the two surgical methods revealed that the recurrence rate significantly decreased as the extent of surgery increased, which is consistent with the reported literature [9, 18, 22, 35].

A limited number of studies have compared the local recurrence and complications rates of patients with and without pathologic fractures due to GCT [3, 4, 6, 21, 26]. Moreover, no studies have compared prognostic outcomes based on the different degrees of pathologic fracture due to GCT. The existing data provide evidence to support that there is no significant difference in the recurrence-free survival rates between the fracture group and nonfracture group [10, 11, 34]. However, our results indicated that the simple fracture group had significantly higher recurrence-free survival rates than the complex fracture group. The overall recurrence rate was 15.4% (20/130) in our study, which were lower than those reported in literatures [6, 9, 11, 12, 14, 21, 34, 38]. The reasons we considered to explain this difference were mainly related to the surgical methods, and fracture itself is an important factor for surgery that determines the surgical extent. Perhaps the results were also affected by the follow-up data obtained via telephone, as it has low reliability and may not exactly represent the true results for local recurrence.

Only few articles have reported on complications after surgical treatments for cases of GCT specifically with pathologic fracture [3, 18, 27]. In the literature, complications were even more frequent after resection and reconstruction with prostheses than after joint salvage. Heijden et al. pointed out in their study that the major complication rate was higher after resection than after curettage (16% versus 4%). Although we did not evaluate the complication rates after the treatment of GCT in patients without pathologic fracture in this study, our results indicated that the group with simple fractures had lower complication rates than the group with complex fractures [4]. We consider this to be due to the fact that resection and reconstruction with joint replacement is performed more often in patients with complex fractures.

The number of studies assessing functional outcomes after treatment for GCT with scoring systems are also limited in the literature. Available reports indicate that better functional outcomes can be achieved with joint salvage than with resection [4, 11, 21, 39]. We found that the group with simple fractures had better outcomes, with higher MSTS and TESS scores, than the group with complex fractures. We consider this difference to be caused by the different surgical methods used between the two groups. However, Prosser et al. found the mean functional scores were similar after curettage and primary resection in their research [40].

The limitations of our study include the following: (1) Patients were recruited from multiple centres, and the quality control methodology was not unified. (2) During follow-up, we found that the compliance of patients was poor; therefore, we used telephone calls instead of outpatient visits to complete our follow-up for some patients. The low recurrence and complication rates might have some relations with our follow-ups adopted. (3) Even though our response rate during follow-up was as high as 86.1%, it sorely stands for the features of our study group.

## Conclusion

This retrospective multicentre study identified several features of GCT patients with pathologic fractures, which could provide new insights into treating pathologic fractures due to GCT. Our classification of “simple fracture” and “complex fracture” could guide decisions regarding the best surgical method for lesions in patients who have giant cell tumour around the knee with different degrees of fracture. We believe pathologic fracture is a clinical concept rather than a radiologic concept. The operation method should be chosen according to the difficulty of the operation itself and after the assessment of other indicators with comprehensive consideration.

## Data Availability

The datasets generated and/or analysed during the current study are not publicly available because the data were from the Information Technology Department of GTOC, who applied restrictions on the data’s public availability. The datasets however are available from the corresponding author on reasonable request and with permission of the Information Technology Department.

## Abbreviations

GCT: Giant cell tumour
GTOC: The giant cell tumour team of China
AP: Anteroposterior
MSTS: The Musculoskeletal tumour society score
TESS: Toronto extremity salvage scoreF
